# At Embryo Implantation Site IL-35 Secreted by Trophoblast, Polarizing T Cells towards IL-35+ IL-10+ IL-4+ Th2-Type Cells, Could Favour Fetal Allograft Tolerance and Pregnancy Success

**DOI:** 10.3390/ijms23094926

**Published:** 2022-04-28

**Authors:** Letizia Lombardelli, Federica Logiodice, Ornela Kullolli, Herman Haller, Chiara Agostinis, Roberta Bulla, Daniel Rukavina, Marie-Pierre Piccinni

**Affiliations:** 1 Department of Experimental and Clinical Medicine, University of Florence, 50134 Florence, Italy; letizia.lombardelli@unifi.it (L.L.); federica.logiodice@unifi.it (F.L.); ornelahysa@hotmail.it (O.K.); 2 Department of Gynecology and Obstetrics, Medical Faculty, University of Rijeka, 51000 Rijeka, Croatia; herman.haller@medri.uniri.hr; 3 Institute for Maternal and Child Health, IRCCS Burlo Garofolo, 34137 Trieste, Italy; cagostinis@units.it; 4 Department of Life Sciences, University of Trieste, 34127 Trieste, Italy; rbulla@units.it; 5 Department of Physiology and Immunology, Medical Faculty, University of Rijeka, 51000 Rijeka, Croatia; rimed@hazu.hr

**Keywords:** IL-35, IL-4, IL-10, pregnancy, Th2 cells, Treg cells, ectopic pregnancy, implantation, p35, trophoblast

## Abstract

We investigated the role of rhIL-35, at low concentrations compatible with those produced by human trophoblast cells (less than 1 ng/mL), on human T helper (Th) cell functions and the presence of decidual IL-35-producing Th cells in human pregnancy. We found that human trophoblast cells produced IL-35 but not IL-4 or IL-10. RhIL-35, at concentrations produced by human trophoblasts, polarized T cells towards IL-35+, IL-10+, IL-4+ Th2-type cells and to Foxp3+ EBI3+ p35+ T reg cells producing IL-35 but not IL-10 and IL-4. Moreover, rhIL-35 at low concentrations did not suppress the proliferation of Th cells but stimulated IL-4 and IL-10 production by established Th clones. In particular, Th1-type clones acquired the capacity to produce IL-4. In addition, purified human trophoblast cell supernatants containing IL-35 upregulated IL-4 and IL-10 production by Th clones. Finally, IL-35+, IL-10+, IL-4+ Th2-type cells, which were found to be induced by low concentrations of IL-35 compatible with those produced by human trophoblasts, are exclusively present in the decidua of a successful pregnancy and at the embryo implantation site, suggesting their stringent dependence on trophoblast cells. Thus, the proximity of Th cells to IL-35-producing trophoblasts could be the determining factor for the differentiation of IL-35+, IL-10+, IL-4+ Th2-type cells that are crucial for human pregnancy success.

## 1. Introduction

The fetus is considered as a semi-allograft that resides in an immune-competent mother. The immune system of the mother, tolerating the allogeneic fetus, sustains the pregnancy.

In fact, the role of CD4+ Th2 cells and Treg cells is crucial for the success of pregnancy. While Th1 cells (in particular IFN-γ) play a role in acute allograft rejection [1,2,3,4], Th2 cells (producing IL-4 and IL-10) and CD4+ CD25+ Foxp3+ T reg cells (producing IL-10 and TGF beta), by inhibiting the Th1 responses, act to enhance allograft tolerance [5,6]. In pregnancy deciduae, Th1 cells have been associated with fetal semi-allograft rejection leading to spontaneous abortion [7], whereas decidual Th2 cells and Treg cells, responsible for fetal semi-allograft tolerance, have been shown to be crucial for successful pregnancy [7,8,9,10,11,12].

More recently, other T-cell cytokines have been involved in fetal semi-allograft tolerance and rejection. In fact, IL-17, when produced alone or together with IFN-γ by T cells, could be deleterious for pregnancy. However, when it is produced in association with Th2-type cytokines, it could turn out to be beneficial for pregnancy. Accordingly, a prevalence of Th17/Th2 cells (producing IL-17 and IL-4) has been found in the deciduae of successful pregnancies, whereas Th17 (producing IL-17 only) and Th17/Th1 (producing IL-17 and IFN-γ) cells have been exclusively observed during a spontaneous abortion in the decidua of a women suffering from unexplained recurrent abortion (URA) [11,12]. These Th17/Th2 cells were exclusively present at the embryo implantation site, whereas Th17, Th17/Th1, and Th1 cells were exclusively present away from the implantation site, suggesting that Th17/Th2 cells could have a key role in embryo implantation [11]. Thus, the decidual Th17 cells producing IL-4 seem to exhibit a helpful role in pregnancy [12]. Similarly, very recently a prevalence of CD4+ T cells producing IL-4 and IL-22 was observed in the decidua of a normal pregnancy, in particular at the embryo implantation site, whereas T helper IL-22+ cells, which did not produce IL-4 but produced IFN-γ and IL-17, were prevalent in the decidua of spontaneous abortions in women suffering from URA and were present far from the embryo implantation site [13]. All these findings suggest that Th2-type cytokines, associated or not to IL-17 and IL-22 production by decidua T cells, could be crucial for the fetal allograft tolerance and for the success of human pregnancy [12].

Trophoblast-derived soluble HLA-G5 induces the switch of human T helper cells into the Th2 cells and Th17/Th2 cells necessary for fetal allograft tolerance and successful pregnancy and also for embryo implantation [11,14]. Thus, trophoblast cells could act as effector cells in supporting maternal–fetal tolerance.

We speculated that IL-35, which is secreted by trophoblast cells [15] and is able to regulate immune responses, could participate in maternal–fetal tolerance in human pregnancy by modulating the decidual CD4+ T cells responses. In fact, interleukin-35 (IL-35), which is a heterodimeric cytokine of the IL-12 family, is able to mediate T helper cell suppression using different mechanisms [16,17,18,19,20].

IL-35 consists of two subunits, an IL-12 α chain (p35) and an Epstein–Barr virus-induced gene 3 (EBI3) β chain. Unlike the other IL-12 family members, it signals through four unconventional receptors: IL-12Rβ2–IL-27Rα, IL-12Rβ2–IL-12Rβ2, IL-12Rβ2–gp130, and gp130–gp130 [19,21].

In mice and humans, naïve T cells stimulated in the presence of IL-35 are not only suppressed but also converted into an IL-35-producing regulatory population termed iTr35 cells [16,22]. These iTr35 cells are phenotypically and functionally distinct from the other known induced regulatory populations (iTregs and Tr1) in that iTr35 cells do not express Foxp3 and suppress exclusively through IL-35 and independently of IL-10, TGFβ, CTLA-4, or any other known Treg suppressive mediators. In fact, the transcription profiling exhibited by iTr35 cells is CD4+ Foxp3− Ebi3+ p35+ IL-10−TGFβ- [16,22]. The human iTr35 cells, induced by IL-35 and producing IL-35, are able to suppress human T-cell proliferation through IL-35, which is also able to inhibit the activities of a range of autoimmune disorders and to mediate infection and cancer tolerance [21,23,24,25]. T-cell suppression and iTr35 induction is mediated through an IL-12Rβ2-gp130 heterodimeric receptor that drives the formation of STAT1–STAT4 heterodimers, which in turn bind to unique sites in the Ebi3 and IL-12α promoters [25].

IL-35 could suppress T helper cells functions not only by inducing iTr35 cells but also by expanding Treg cells. In fact, exposure to IL-35 has been shown to induce the proliferation of a population of CD4+ CD25+ Foxp3+ T cells [26]. IL-35 is able to potently inhibit the CD4+ Th1 and Th17 cells through the expansion of these Treg cells producing IL-10 [18,27]. These IL-35-expanded Tregs expressing IL-10 are able to suppress effector T-cell proliferation and to contribute to IL-35 inhibitory effects in autoimmune disorders characterized by Th1/Th17 inflammation [16,18,26,28,29]. In addition, IL-35 could also stimulate Th2 cell functions. In fact, IL-35 induces IL-10 production in vitro by murine Th2 cells [30]. IL-35 also plays key role in modulating the ratio of M1/M2 macrophages, inducing the tolerogenic phenotype on dendritic cells contributing to the Th2 polarization of T cells [31]. However, none of the studies previously published on IL-35 effects on the T cell responses used low concentrations of IL-35. They were all performed using very high levels of IL-35 (from 10 ng/mL to 100 ng/mL), which are 10- to 100-fold higher than those produced by the human trophoblast cells.

Moreover, the role of IL-35 on allograft tolerance has been suggested by injecting interleukin-35-modified mesenchymal stem cells (IL-35-MSCs) in mice. These cells induce a suppression of cardiac allograft rejection. IL-35-MSCs show a strong immunosuppressive ability compared to MSCs by reducing the percentage of Th17 cells, increasing the proportion of CD4+, Foxp3+ T cells, and regulating Th1/Th2 balance in heart transplant mice. These findings showing that IL-35-MSCs have more advantages than MSCs in inhibiting graft rejection suggest that IL-35 could be key factor for allograft tolerance [32,33].

Given the immunosuppressive role of IL-35 and its expression and production by trophoblast cells [15], we speculated that IL-35 secreted by trophoblast cells may participate in maternal–fetal tolerance by modulating T helper cell proliferation and differentiation. For this reason, we studied, for the first time, the role of IL-35, at concentrations compatible to those produced by human trophoblasts, on Th2, Th1, and Th17 cell cytokine profiles and on T cell proliferation and also studied the direct effect of human trophoblast cells on the T cell cytokine profile.

Only one study, which was performed in mice, has analyzed the expression of IL-35 at the fetal–maternal interface and the importance of IL-35 for successful pregnancy, showing that the expression of IL-35 mRNA and protein is downregulated in the placenta of females of the CBA/J × DBA/2 J mating group, which is associated with spontaneous abortion, compared to females of the CBA/J × BALB/C mating group, which is associated with successful pregnancy [34]. In humans, the fact that IL-35 could favor the success of human pregnancy has been suggested only at the serum level by two studies reporting that the levels of IL-35 decreased in the plasma of women with a history of idiopathic recurrent pregnancy loss compared to fertile controls [35,36]. Here, we examined, for the first time in human pregnancy at the decidual level and at embryo implantation site of ectopic pregnancy, CD4+ T cells producing IL-35. In fact, we analyzed IL-35-producing CD4+ T cells at the embryo implantation site and far from the implantation site of ectopic tubal pregnancies from the decidua of the same women with ectopic pregnancy, where the implantation site is not present in the decidua but in the fallopian tube, and from the decidua of women with normal pregnancy with a regular embryo implantation site.

## 2. Results

### 2.1. Effect of rhIL-35 on the Produced Cytokine and on the Cytokine mRNA Expression of SK-Specific T-Cell Lines

**Figure 1:**
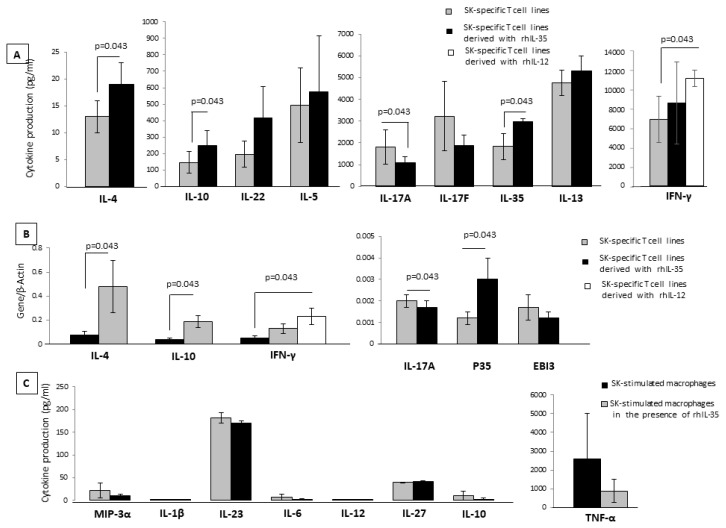
Effect of rhIL-35 on the cytokine production and on the cytokine mRNA expression of SK-specific T-cell lines and on the cytokine profile of macrophages. PBMCs from 10 different donors were stimulated with SK in the absence or presence of rhIL-35 at 300 pg/mL. (**A**) The levels of IL-4, IL-13, IL-10, IL-22, IL-17A, IL-17F, IL-35, and IL-5 in response to PMA plus anti-CD3 mAb in the T-cell lines specific for SK generated in the presence and absence of rh IL-35 (300 pg/mL) were measured with a multiplex bead-based assay. (**B**) T-cell lines specific for SK generated in the presence or absence of rhIL-35 (300 pg/mL) in bulk PBMC cultures were also analyzed with RT-PCR for the ability to express mRNA for IFN-γ, IL-4, IL-10, IL-17A, p35, and EBI3 in response to PMA plus anti-CD3 mAb. (**C**) Purified macrophages from PBMC obtained from five healthy donors stimulated for 5 days with the antigen SK in the absence or in the presence of rh IL-35 (300 pg/mL). MIP-3α, IL-1β, IL-23, IL-6, IL-12, IL-27, IL-10, and TNF-α were measured in the supernatants of macrophages with a multiplex bead-based assay.

Ten T-cell lines specific for SK generated in the presence and absence of rh IL-35 in bulk PBMC cultures were analyzed for the ability to produce IFN-γ, IL-4, IL-13, IL-10, IL-22, IL-17A, IL-17F, IL-35, and IL-5 in response to PMA plus anti-CD3 mAb with a multiplex bead-based assay. When the T-cell lines were generated with rIL-12, the levels of IFN-gamma increased (*p* = 0.043) (Figure 1A), suggesting that the culture conditions were satisfactory for the modulation of T-cell lines. As shown in Figure 1, the production of the two Th2-type cytokines, IL-4 and IL-10, and IL-35 production were significantly increased, whereas the production of the Th17-type cytokine, IL-17A, was significantly decreased when SK-specific T-cell lines were generated in the presence of IL-35 compared to SK-specific T-cell lines generated in the absence of IL-35. No significant effect of rh IL-35 on the production of IFN-gamma was observed (Figure 1A), and no effects of rhIL-35 on the production of the other two Th17-type cytokines, IL-17F and IL-22, or the other two Th2-type cytokines, IL-13 and IL-5, were observed (Figure 1A).

T-cell lines specific for SK generated in the presence or absence of rhIL-35 in bulk PBMC cultures were also analyzed with RT-PCR for the ability to express mRNA for IFN-γ, IL-4, IL-10, IL-17A, p35, and EBI3 in response to PMA plus anti-CD3 mAb. When the T-cell lines were generated with rIL-12, the levels of mRNA for IFN-γ increased (*p* = 0.043) (Figure 1B), suggesting that the culture conditions were satisfactory for the modulation of T-cell lines. According to the previously obtained results (Figure 1A), IL-4, IL-10, and p35 RNA expression was significantly increased, whereas the expression of mRNA for IL-17A was significantly decreased when SK-specific T-cell lines were generated in the presence of rhIL-35 compared to SK-specific T-cell lines generated in the absence of rhIL-35. The expression of mRNA for IFN-γ and EBI3 was not modified when SK-specific T-cell lines were generated in the presence or in the absence of rh IL-35 (Figure 1B).

These results indicate that rhIL-35 at low concentrations stimulates IL-4, IL-10, and IL-35 production but inhibits IL-17A production by antigen-specific T cells.

### 2.2. Effect of rh IL-35 on the Cytokine Profile of SK-Activated Macrophages Derived from Peripheral Blood Monocytes

The negative effect of rhIL-35 on the Th17-type cytokine (IL-17A) production and its positive effect on the production of the two Th2-type cytokines (IL-4 and IL-10) of antigen-specific T-cell lines could be due to the indirect modulating effects of rhIL-35 on APCs, which present SK to the T cells when the antigen-specific T-cell lines were induced. We found that the levels of MIP-3α, IL-1β, IL-23, IL-6, IL-12, IL-27, IL-10, and TNF-α produced by SK-stimulated macrophages cultured in the presence of rh IL-35 were not significantly different than those of SK-stimulated macrophages cultured in the absence of rhIL-35 (Figure 1C). Thus, the decreased levels of IL-17A produced by SK-specific T-cell lines in response to rhIL-35 seem to be independent of the stimulating effect of rhIL-35 on IL-27, a negative regulator of Th17 cell differentiation and/or of the suppressing effects of rhIL-35 on the macrophage IL-23 and IL-1β productions that are essential for Th17 development. Moreover, the decreased levels of IL-17A produced by SK-specific T-cell lines in response to rhIL-35 also seem to be independent of the suppressing effect of rhIL-35 on the trafficking of Th17 regulated by the macrophage MIP-3α production. In addition, the increased levels of Th2-type cytokines produced by SK-specific T-cell lines in response to rhIL-35 appeared to be independent of the stimulating effect of rhIL-35 on IL-10 macrophage production. The fact that rhIL-35 had no effect on the macrophage IL-12 production could explain the fact that rhIL-35 had no effect on the SK-specific T-cell line IFN-γ production.

Thus, the stimulating effect of rhIL-35 on IL-4 and IL-10 production and the suppressing effect of rh IL-35 on the IL-17 production of SK-specific T-cell lines seem to be due to the direct action of rhIL-35 on T cells and independent of the indirect effects of rhIL-35 on the APCs, which are essential in the PBMC fraction to present SK to the SK-specific T cells.

### 2.3. IL-35 Favors the Development of Th2 (IL-4+ IL-10+) Cells Producing IL-35

To identify the T-cell type(s) responsible for the production of IL-35, IL-10, and IL-4 by SK-specific T-cell lines in response to rhIL-35, T-cell blasts from SK-specific T-cell lines generated in the absence or in the presence of rhIL-35 derived from the peripheral blood of two different donors were cloned under limiting dilution conditions. The SK-specific T-cell clones derived from the SK-specific T-cell lines generated with rhIL-35 and without rhIL-35 were assessed for their cytokine profile in response to stimulation with PMA plus anti-CD3 mAb.

In total, 68 CD4+ T-cell clones were derived from the SK-specific T-cell lines of which 27 CD4+ SK-specific T-cell clones were generated from SK-T-cell lines derived in the absence of rhIL-35 (39.7%) and 41 T-cell clones were generated in the presence of rhIL-35 (60.3%).

**Figure 2:**
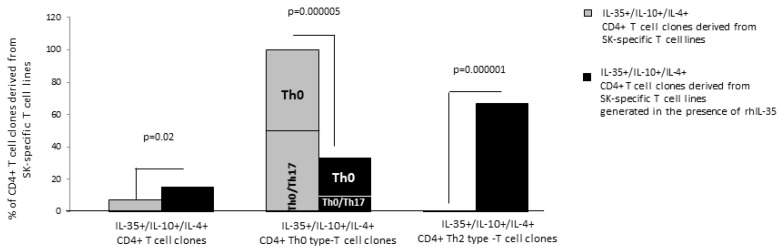
rhIL-35 favors the development of IL-35 + IL-10 + IL4 + Th2-type cells in SK-specific CD4+ T-cell lines. T-cell blasts from SK-specific T-cell lines generated in the absence or in the presence of rhIL-35 (300 pg/mL) derived from the peripheral blood of two different donors were cloned under limiting dilution conditions. The SK-specific T-cell clones derived from the SK-specific T-cell lines generated with rhIL-35 and without rhIL-35 were assessed for their cytokine profiles in response to stimulation with PMA plus anti-CD3 mAb. The percentage of CD4+ T-cell clones, Th2-type T-cell clones, and Th0-type T-cell clones producing IL-35, IL-10, and IL-4 was determined.

The percentage (6/41 = 14.5%) of CD4+ T-cell clones producing IL-4, IL-10, and IL-35 (IL-35+/IL-10+/IL-4+ CD4+ T-cell clones) was significantly higher when CD4+ T-cell clones derived from SK-T-cell lines were generated in the presence of rhIL-35 than when they were derived in the absence of rhIL-35 (2/27 = 7.5%) (*p* = 0.02) (Figure 2). Moreover, the Th2-type IL-35+/IL-10+/IL-4+ CD4+ T-cell clones, producing IL-35, IL-10 and IL-4 but not IFN-γ, were exclusively seen when CD4+ T-cell clones were derived from SK-T-cell lines generated in the presence of rhIL-35, whereas no Th2-type IL-35+/IL-10+/IL-4+ CD4+ T-cell clones were observed when CD4+ T-cell clones were derived from SK-T-cell lines generated in the absence of rhIL-35 (*p* = 0.000001) (Figure 2). By contrast, the percentage of Th0-type IL-35+/IL-10+/IL-4+ CD4+ T-cell clones, producing IL-35, IL-10, IL-4, and IFN-γ with or without IL-17A (Th0/Th17 and Th0, respectively) derived from SK-T-cell lines generated in the presence of rhIL-35 decreased compared to the percentage of Th0-type IL-35+/IL-10+/IL-4+ CD4+ T-cell clones derived from SK-T-cell lines generated in absence of rhIL-35 (*p* = 0.000005) (Figure 2).

These results indicate that the Th2-type CD4+ T-cell clones producing IL-35, IL-10, and IL-4, are exclusively generated when APC present the antigen to specific CD4+ T cells in the presence of exogenous IL-35. Therefore, IL-35 could be an inducer of Th2 cells (IL-4+ and IL-10+) producing IL-35.

### 2.4. rhIL-35 Favors the Development of Foxp3 Treg Cells Producing IL-35

To identify another CD4+ T cell subpopulation that is different from the Th2 (IL-4+ IL-10+ IL-35+)-type cell and could be responsible for the production of one or more of the cytokines, IL-35, IL-10, and IL-4 by SK-specific T-cell lines in response to rhIL-35, we analyzed other CD4+ T-cell clones (*n* = 25) derived from SK-specific T-cell lines generated in the absence or in the presence of rhIL-35 (*n* = 35) and obtained from the peripheral blood of two donors.

**Figure 3:**
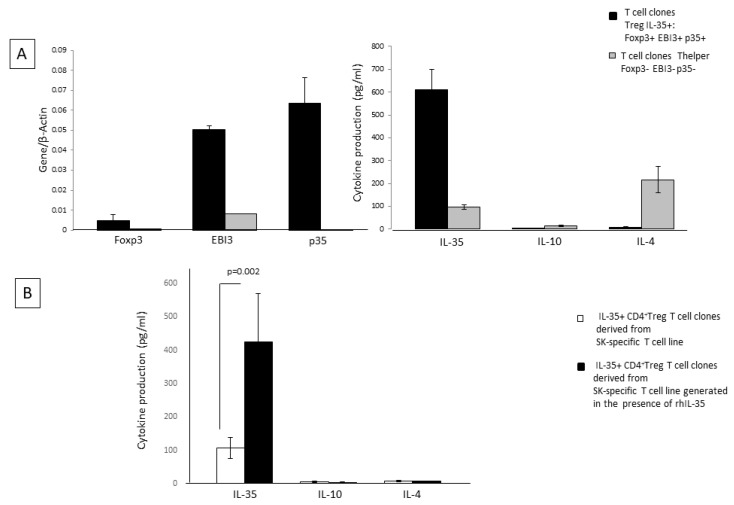
rhIL-35 favors the development of Foxp3 Treg cells producing IL-35 in SK-specific CD4+ T-cell lines. T-cell blasts from SK-specific T-cell lines generated in the absence or in the presence of rhIL-35 (300 pg/mL) derived from the peripheral blood of two different donors were cloned under limiting dilution conditions. (**A**) The derived CD4+ T-cell clones were analyzed with RT-PCR for their ability to express mRNA for Foxp3, EBI3, and p35 and for the ability to produce IL-35, IL-4, and IL-10 in response to PMA plus anti-CD3 mAb. (**B**) The Treg-type clones expressing Foxp3 derived from the SK-specific T-cell lines generated with rhIL-35 and without rhIL-35 were assessed for IL-35, IL-10, and IL-4 production in response to PMA plus anti-CD3 mAb stimulation.

Besides the CD4+T-cell clones derived from SK-specific T-cell lines generated in the presence of rhIL-35 and in the absence of rhIL-35, which did not express mRNA for Foxp3 and p35 but expressed mRNA for EBI3 and produced IL-10, IL-4, and IL-35 (Figure 3A), we found CD4+ T-cell clones expressing Foxp3, EBI3, p35, STAT1, and STAT4 (two well-known transcription factors expressed by regulatory T cells expressing IL-35) and producing IL-35 but not IL-10 or IL-4 (Figure 3A). The percentage of Treg-type IL-35+, IL-10-, IL-4- T-cell clones derived from SK-specific T-cell lines generated in the presence of rhIL-35 was not significantly higher (11/41 = 27%) than the percentage of Treg-type T-cell clones derived in the absence of rhIL-35 (5/27 = 18.5%). However, these Treg clones, derived from SK-specific T-cell lines generated in the presence of rhIL-35, produced levels of IL-35 that are significantly higher than those produced by Treg clones derived from SK-specific T-cell lines generated in the absence of rhIL-35 (*p* = 0.002) (Figure 3B). Thus, rhIL-35 seems to stimulate the production of IL-35 by Foxp3+ Treg cells, which do not produce IL-4 and IL-10 but, does not induce the expansion of the IL-35+ Treg cells.

Thus, IL-35 produced by the SK-T-cell lines generated in presence of exogenous IL-35 seem to derive from the 14.5% of Th2 CD4+ T helper cells producing IL-35 in addition to IL-4 and IL-10 and from the 27% of Foxp3+ Tregs that produce IL-35 but do not produce IL-4 or IL-10 (Figure 3B). The other CD4+ T-cell clones, which represent 58.5% of the CD4+ T-cell clones derived in presence of exogenous IL-35, are mostly conventional Th2 and Th0 cells.

### 2.5. IL-35 Can Modulate the Cytokine Profile of Established CD4+ T-Cell Clones

To provide evidence of the direct effect of rhIL-35 on CD4+T helper cells, we examined the ability of rhIL-35 to act on the cytokine production of 50 established CD4+ T-cell clones.

**Figure 4:**
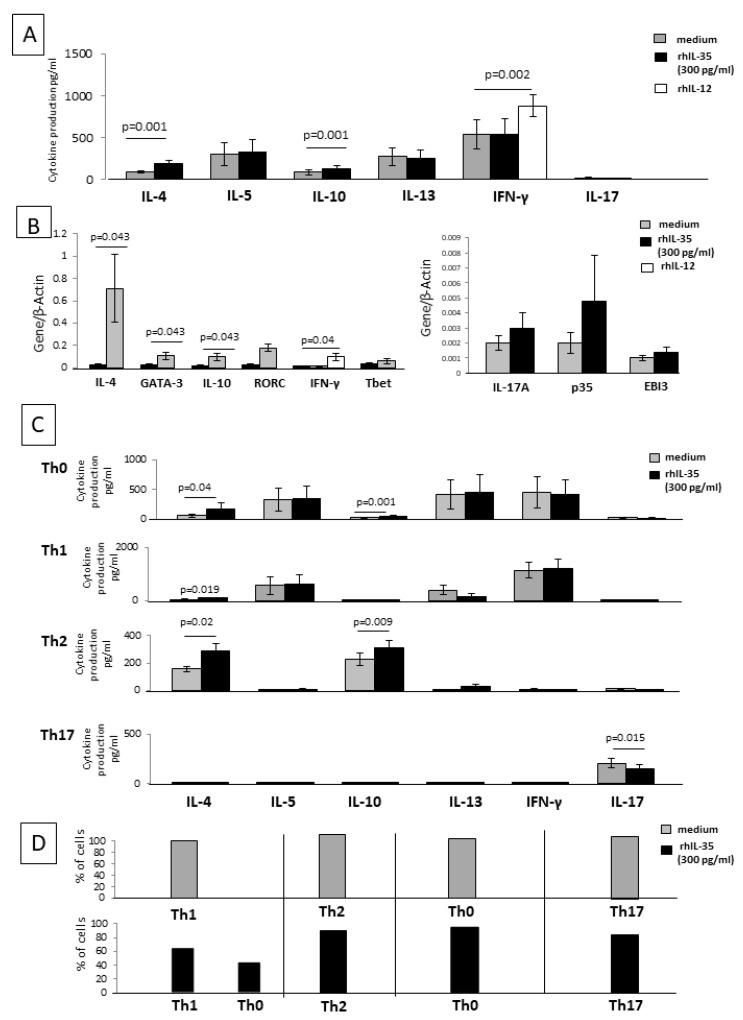
Direct effects of rh IL-35 on the cytokine profile of established CD4+ T-cell clones. (**A**) To provide evidence of the direct effect of rhIL-35 (300 pg/mL) on the cytokine production by T cells, IL-4, IL-5, IL-13, IL-17A, IFN-γ, and IL-10 production were measured with multiplex assays in the supernatant of 15 established T-cell clones stimulated by an immobilized anti-CD3 mAb. (**B**) To provide evidence of the direct effect of rh IL-35 (300 pg/mL) on the mRNA expression of IFN-γ, IL-4, IL-10, IL-17A, GATA3, T-bet, p35, EBI3, and RORC, RT-PCR was performed on five additional established T-cell clones stimulated by an immobilized anti-CD3 mAb. (**C**) To provide evidence of the direct effect of rh IL-35 (300 pg/mL) on different CD4+T cell subpopulations, five additional CD4+ Th1 T-cell clones, five additional CD4+ Th2 T-cell clones, five additional CD4+ Th17 T-cell clones, and five additional CD4+ Th0 2 T-cell clones were stimulated with an insolubilized anti-CD3 monoclonal antibody in the absence or presence of rhIL-35 (300 pg/mL). The levels of IL-4, IL-5, IL-13, IL-10, IL-17A, and IFN-γ were measured in the T-cell clone supernatants by multiplex bead-based assays. (**D**) To provide evidence of the direct effect of rhIL-35 (300 pg/mL) on different CD4+ T cell subpopulations, six additional CD4+ Th1, six additional CD4+ Th2, six additional CD4+ Th17, and six additional CD4+ Th0 T-cell clones were stimulated with an insolubilized anti-CD3 monoclonal antibody in the absence or presence of rhIL-35 (300 pg/mL). The percentages of T-cell clones producing IFN-γ alone (Th1-type T-cell clones); producing IL-4, IL-5, IL-13, and IL-10 (Th2-type T-cell clones); producing IL-17A (Th17-type T-cell clones); and producing IFN-γ, IL-4, IL-5, IL-13, IL-10, and IL-17A (Th0-type T-cell clones) were determined to evaluate the possible cytokine profile modification induced by rhIL-35 on established CD4+ T-cell clones.

We first studied rhIL-35 effects on the cytokine and transcriptional factor mRNA expression of 20 established CD4+ T-cell clones. CD4+ T-cell clones derived from the peripheral blood of healthy donors were cultured in the presence of recombinant human IL-12 (rhIL-12), a Th1 inducer that acts as a control of the functional activity of T-cell clones. When the twenty CD4+ T-cell clones were cultured with rhIL-12, the levels of IFN-γ and mRNA for IFN-γ increased (*p* = 0.002 and *p* = 0.04 respectively) (Figure 4A,B), suggesting that the culture conditions were satisfactory for the modulation of CD4+ T-cell clones.

When 15 of the 20 established CD4+ T-cell clones with defined cytokine profiles were cultured in the absence of any other cell type and stimulated with an insolubilized anti-CD3 monoclonal antibody in the absence or presence of rhIL-35, the levels of IL-5, IL-13, IL-17, and IFN-γ were not significantly modified in the presence of rhIL-35. In contrast, the levels of IL-4 and IL-10 produced by the CD4+ T-cell clones were significantly increased in the presence of rhIL-35 compared to those found in the absence of rhIL-35 (Figure 4A).

A real-time RT-PCR analysis was used to determine the influence of rhIL-35 on IFN-γ, IL-4, IL-10, IL-17A, GATA3 (a Th2-specific transcription factor), T-bet (a Th1-restricted transcription factor), ROR-C (a Th17-specific transcription factor), and p35 and EBI3 (the two subunits of IL-35) mRNA expression of five additional CD4+T-cell clones stimulated with an immobilized anti-CD3 monoclonal antibody without or with rhIL-35 (Figure 4B). The CD4+ T-cell clones expressed higher levels of IL-4, IL-10, and GATA3 mRNA in the presence of rhIL35 compared to the control T-cell clones, whereas the levels of transcripts for IFN-γ, IL-17A, ROR-C, T-bet, p35, and EBI3 were not modified by the presence of rhIL-35 in the culture of the CD4+ T-cell clones (Figure 4B).

These results confirm that rhIL-35 stimulates the production of Th2-type cytokines IL-4 and IL-10 and the related transcription factor GATA-3 but suggest that rhIL-35 has no effect on the IL-35 production of stabilized CD4+ T-cell clones. Thus, IL-35 could be able to modify the cytokine profile of established CD4+ T-cell clones.

To verify the direct effect of rhIL-35 on the different CD4+T cell subpopulations, five Th1 clones, five Th2 clones, five Th17 clones, and five Th0 clones were stimulated with insolubilized anti-CD3 monoclonal antibody in the absence or presence of rhIL-35 (Figure 4C) and were analyzed for IL-4, IL-5, IL-10, IL-13, IL-17A, and IFN-γ production.

In the presence of rhIL-35, the levels of IL-5, IL-13, IFN-γ, and IL-17A produced by the Th0 cells were not modified, whereas the levels of IL-4 (*p* = 0.04) and IL-10 (*p* = 0.001) were significantly increased in the presence of rhIL-35 compared to those found in the absence of rhIL-35 (Figure 4C).

In the presence of rhIL-35, the levels of IL-5, IL-13 (described to also be produced by Th1 cells [37]), IFN-γ, IL-17A, and IL-10 produced by Th1 cells were not significantly modified. More importantly, the levels of IL-4 produced by Th1 cells were significantly increased in the presence of rhIL-35 (*p* = 0.019), whereas they were unsubstantial in the absence of rhIL-35 (Figure 4C).

The levels of IL-4 and IL-10 produced by Th2 cells were significantly increased in the presence of rhIL-35 compared to those found in the absence of rhIL-35 (*p* = 0.02, *p* = 0.009, respectively) (Figure 4C).

The production of IL-4, IL-5, IL-10, IL-13, and IFN-γ by Th17 cells remained unsubstantial in the presence of rhIL-35, whereas the levels of IL-17A by Th17 cells were significantly decreased in the presence of rhIL-35 compared to those found in the absence of rhIL-35 (*p* = 0.015) (Figure 4C).

These data confirm the negative effects of rhIL-35 on the production of IL-17A by Th17 cells, as previously shown with the SK-specific T-cell lines, and support the finding that IL-4 and IL-10 production is stimulated by rhIL-35 in Th2 and Th0 cells, which were already able to produce these cytokines. In addition, our results highlight the possible induction of the production of IL-4, but not of IL-10, by Th1 cells in response to rhIL-35.

Moreover, to verify the direct effect of rhIL-35 on the different CD4+T-cell clones, six additional Th1 clones, six Th2 clones, six Th17 clones, and six Th0 clones were stimulated with insolubilized anti-CD3 monoclonal antibody in the absence or presence of rhIL-35 (Figure 4D) and the percentage of T-cell clones changing their cytokine pattern was analyzed. Four of the six (67%) Th1 clones remained Th1 in the presence of rhIL-35, whereas two of them (33%) became a Th0 clone (which continued to produce IFN-γ, but also produced IL-4 in response to rhIL-35) (Figure 4D).

All six Th2 clones remained Th2 as the six Th17 clones remained Th17 and the Th0 clones remained Th0 (Figure 4D).

Thus, rhIL-35 is not only able to induce the differentiation of CD4+T cells towards a Th2-type profile when APCs present the antigen to CD4+ T cells but is also able to modify the cytokine profile of established CD4+ T-cell clones. In particular, 33% of Th1-type clones acquired the ability to produce IL-4 in the presence of rhIL-35.

### 2.6. Trophoblast Cells, through the Spontaneous Production of IL-35, Stimulate IL-4 and IL-10 Production by CD4+ T Helper Cells

**Figure 5:**
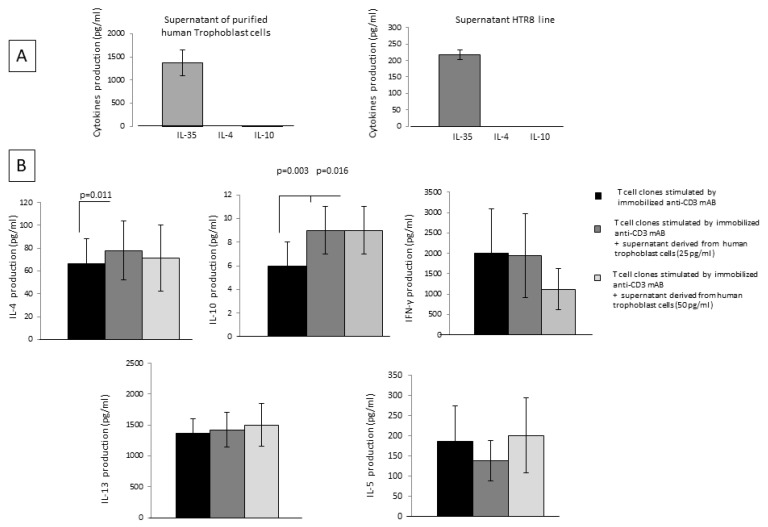
Trophoblast cells, through the spontaneous production of IL-35, stimulate IL-4 and IL-10 production by CD4+ T helper cells. (**A**) IL-35 was measured with a bead-based assay in the supernatant of 16 specimens of isolated human first-trimester cytotrophoblast cells and in the supernatant of 3 specimens from the trophoblast cell line HTR8. (**B**) Fourteen CD4+ T-cell clones derived from the peripheral blood of healthy subjects stimulated with immobilized anti-CD3 mAb were cultured for 36 h in the absence and in the presence of IL-35 derived from four isolated human first-trimester cytotrophoblast cell supernatants at two different final concentrations, 25 pg/mL and 50 pg/mL.

IL-35 was measured with a bead-based assay in the supernatant of 16 specimens of isolated human first-trimester cytotrophoblast cells and in the supernatant of 3 specimens from the trophoblast cell line HTR8 (Figure 5A). The levels of IL-35 measured in specimens of purified primary trophoblast were 1377 ± 278 pg/mL, and the levels of IL-35 measured in HTR8 were 217 ± 15 pg/mL. In addition, isolated human first-trimester cytotrophoblast cells and HTR8 cells were not able to produce IL-4 or IL-10 (Figure 4A). These results show that trophoblast cells produce IL-35 spontaneously at the human fetal-maternal interface but not IL-10 or IL-4.

Moreover, fourteen CD4+ T-cell clones derived from the peripheral blood of healthy subjects stimulated with immobilized anti-CD3 mAb were cultured for 36 h in the absence and in the presence of IL-35 derived from four isolated human first-trimester cytotrophoblast cell supernatants at two different final concentrations, 25 pg/mL and 50 pg/mL (Figure 5B).

IL-4 production was significantly increased when CD4+ T-cell clones were cultured in the presence of trophoblast cell supernatants containing 25 pg/mL of IL-35 (but not IL-4 and IL-10) (*p* = 0.011), compared to T-cell clones cultured without trophoblast supernatants. Moreover, IL-10 was significantly increased when CD4+ T-cell clones were cultured in the presence of trophoblast cell supernatants containing 25 pg/mL of IL-35 (*p* = 0.003) and 50 pg/mL of IL-35 (*p* = 0.016) (but not IL-4 and IL-10) compared to T-cell clones cultured without trophoblast supernatants. No significant difference was observed for the levels of the other Th2-type cytokines, IL-13 and IL-5, and for IFN-γ productions by CD4+ T-cell clones in the presence of trophoblast cell supernatants and T-cell clones cultured without trophoblast supernatant (Figure 5B).

These results suggest that human extravillous trophoblast cells do not produce IL-4 or IL-10 but are able to spontaneously produce IL-35 to stimulate the production of IL-4 and IL-10 by CD4+ T cells.

### 2.7. RhIL-35, at Low Concentrations Compatible with Those Produced by Human Primary Trophoblasts, Does Not Inhibit CD4+ T-Cell Clones Proliferation

**Figure 6:**
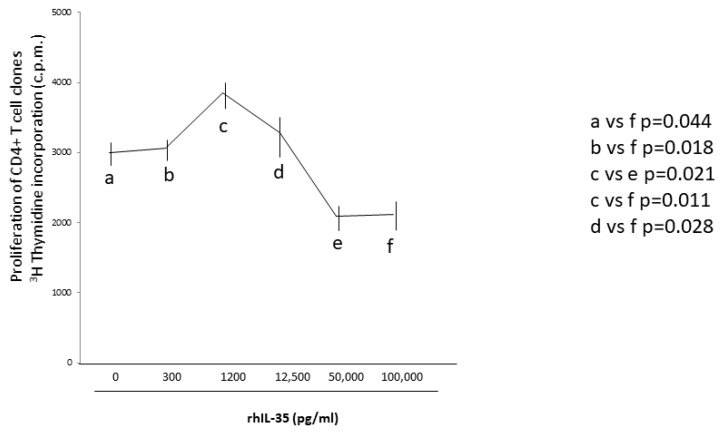
RhIL-35, at low concentrations compatible with those produced by human primary trophoblasts, does not inhibit the CD4+ T-cell clone proliferation, but at high concentrations it inhibits T-cell clone proliferation. Nine additional CD4+ T-cell clones generated from the peripheral blood of healthy subjects were stimulated with immobilized anti-CD3 mAb in the absence and in the presence of different concentrations of rhIL-35 (range 100 pg/mL- 100 ng/mL), and the proliferative activity of these T-cell clones was evaluated by ^3^H thymidine incorporation (c.p.m.).

To assess if rhIL-35 could regulate T helper cell activity not only by stimulating Th2-type cytokine production but also by inhibiting the T helper cell proliferation, nine additional CD4+ T-cell clones generated from the peripheral blood of healthy subjects were stimulated with an immobilized anti-CD3 mAb in the absence and in the presence of different concentrations of rhIL-35, and the proliferative activity of these T-cell clones was evaluated (Figure 6). We found a statistically significant inhibition of the CD4+ T-cell clone proliferation only at very high concentrations of rhIL-35 (100 ng/mL) compared to the proliferation of the same CD4+ T-cell clones cultured without rhIL-35 (Figure 6). Interestingly, compared to the proliferation of the CD4+ T-cell clones cultured without rhIL-35, we did not find a statistically significant inhibition of the CD4+ T-cell clone proliferation at IL-35 concentrations compatible with those spontaneously produced by human primary trophoblast cells and the trophoblast cell line HTR8 (from 300 to 1200 pg/mL) (Figure 6). The proliferative response of the CD4+ T-cell clones in response to concentrations of IL-35 compatible with those produced by human primary trophoblast cells and the trophoblast cell line HTR8 was statistically lower than the proliferative response of the CD4+ T-cell clones in response to 100 ng/mL of rhIL-35 (Figure 6).

These results suggest that the low concentrations of IL-35 produced by trophoblast cells are unable to suppress the proliferative response of CD4+ T helper cells, while very high concentrations of IL-35, which are not compatible with IL-35 trophoblast production, could be able to inhibit CD4+ T cell proliferation.

### 2.8. Th2-Type CD4+ T Cells Producing IL-35, IL-10, and IL-4 Are Exclusively Present at Embryo Implantation Site

**Figure 7:**
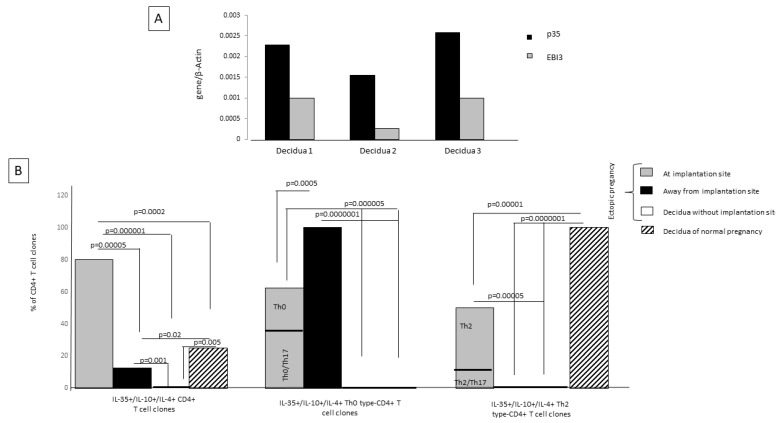
Th2 CD4+ T cells producing IL-35, IL-10, and IL-4 are exclusively present at the embryo implantation site and in the decidua of normal human pregnancy. (**A**) mRNA expression for p35 and EBI3 was evaluated in three decidua specimens of first-trimester normal pregnancy. (**B**) Seventy CD4+ T-cell clones were generated from each of these tissues: at the embryo implantation site and far from the implantation site of ectopic tubal pregnancies, from the decidua of the same women with ectopic pregnancy, where the implantation site was not present in the deciduae, but in the fallopian tube and from the deciduae of women with normal pregnancy with an embryo implantation site. The percentages of CD4+ T-cell clones, Th2-type T-cell clones, and Th0-type T-cell clones producing IL-35, IL-10, and IL-4 were determined in the different tissues.

When observing the expression of mRNA for p35 and EBI3, the two co-expressed subunits of IL-35, in three human decidual specimens obtained from women with normal pregnancy during the first trimester of gestation, we found that IL-35 is expressed at the decidual level (Figure 7A). Despite Ebi3 and p35 mRNA having been shown to be co-expressed in trophoblast cells, they were not detected in first-trimester decidua stromal cells [15]. These results suggest that IL-35 expression at the decidual level is derived from decidual T cells and not from stromal cells. For this, we derived and analyzed T-cell clones from first-trimester deciduae of normal pregnancy and from the embryo implantation site. We generated 70 CD4+ T-cell clones from ectopic tubal pregnancies at the implantation site and far from the implantation site and also from the deciduae of the same women with ectopic pregnancy, where the implantation site was not present in the decidua but in the fallopian tube, and from the deciduae of women with normal pregnancy with an implantation site.

We evaluated the percentage of IL-35+, IL-10+, IL-4+, CD4+ T-cell clones among all CD4+ T-cell clones derived from the implantation site of the embryo, far from the implantation site in the same fallopian tube, from the decidua of the same woman suffering from ectopic pregnancy, and from the decidua of a normal pregnancy. We also evaluated the percentages of IL-35+, IL-10+, IL-4+ Th2-type CD4+ T-cell clones and IL-35+, IL-10+, IL-4+ Th0-type CD4+ T-cell clones (producing IFN-γ and/or IL-17 A also) among the generated IL-35+, IL-10+, IL-4+, CD4+ T-cell clones (Figure 7B).

At the implantation site, the percentage of tubal IL-35+/IL-10+/IL-4+ CD4+ T-cell clones (80%) was higher than the percentage of tubal IL-35+/IL-10+/IL-4+ CD4+ T-cell clones derived far from the implantation site (12.8%) (*p* = 0.00005) and from the ectopic pregnancy T-cell clones derived from decidua without embryo implantation (0%) (*p* = 0.000001). Interestingly, at the implantation site the percentage of IL-35+/IL-10+/IL-4+ CD4+ T-cell clones (80%) was also higher than the percentage of those T-cell clones derived from the deciduae of successful pregnancies (25%) (*p* = 0.0002) (Figure 7B), indicating that the IL-35+/IL-10+/IL-4+ CD4+ T cells are extensively present at the embryo implantation site.

In ectopic pregnancies, the IL-35+, IL-10+, IL-4+ Th2-type CD4+ T-cell clones are exclusively present at the implantation site and are absent far from the implantation site (*p* = 0.00001) and in the decidua without an implantation site (*p* = 0.0000001). In successful pregnancies the percentage of IL-35+, IL-10+, IL-4+ Th2-type CD4+ T-cell clones is significantly higher compared to the percentage of IL-35+, IL-10+, IL-4+ Th2-type CD4+ T-cell clones at the implantation site of ectopic pregnancies (*p* = 0.00001) (Figure 7).

In ectopic pregnancies the IL-35+, IL-10+, IL-4+ Th0-type CD4+ T-cell clones, which also produce the proinflammatory cytokine IFN-γ, are extensively present far from the implantation site, decreased at the implantation site (*p* = 0.0005), where they also produce IL-17, and are absent in the decidua without an implantation site and in the deciduae of normal pregnancies (Figure 7B).

IL-35+/IL-10+/IL-4+ CD4+ T-cell clones (IL-35+/IL-10+/IL-4+ Th2-type CD4+ T-cell clones and IL-35+/IL-10+/IL-4+ CD4+ Th0-type T-cell clones) were absolutely absent from the decidua without any implantation site (Figure 7), which is very far from the tubal implantation site. However, in the fallopian tube far from the tubal implantation site, but not so far compared to the decidua without any implantation, some IL-35+/IL-10+/IL-4+ CD4+ T-cell clones (12.8%) could be found, but they were IL-35+/IL-10+/IL-4+ Th0 cells that also produced IFN-γ. None of them were IL-35+, IL-10+, IL-4+ Th2-type CD4+ T-cell clones.

All these results indicate that the presence of IL-35+, IL-10+, IL-4+ Th2-type CD4+ T-cells is stringently dependent on the presence of an implanted embryo, which is able to produce IL-35. The proximity of CD4+ T cells to IL-35-producing trophoblasts could be a determining factor for the differentiation of CD4+ T cells towards IL-35+, IL-10+, IL-4+ CD4+ T cells and, in particular, towards IL-35+, IL-10+, IL-4+ Th2-type cells.

## 3. Discussion

The fetus, which expresses paternal HLA-C molecules, is considered to be a semi-allograft that resides in an immune-competent mother. The immune system of the mother, tolerating the allogeneic fetus, could sustain a successful pregnancy. In fact, the role of Th2, Th2/Th17 cells, and Treg cells in inhibiting the Th1 and Th17/Th1 responses responsible for fetal rejection is crucial for the success of pregnancy [7,8,9,11,12,38]. We speculated that the IL-35 secreted by trophoblast cells could participate in the maternal–fetal tolerance in human pregnancy by modulating the decidual CD4+ T cells responses.

We found that purified first-trimester human extravillous trophoblasts and a first-trimester trophoblast cell line, HTR8, produce IL-35, but they did not produce the Th2-type cytokines, IL-4 and IL-10 (Figure 5). Our data, showing the constitutive production of IL-35 by human trophoblasts, are in agreement with reports showing that Ebi3 and p35 mRNA expression is observed in trophoblast cells and that IL-35 is detected in the supernatants of HTR8 cells [15,34].

IL-35 has already been shown to play a potent immunosuppressive role [23] by directly suppressing effector T cell proliferation by inhibiting the functions of Th1 and Th17 cells through the expansion of Treg cells producing IL-10 [27] and by generating a potent population of IL-35-expressing inducible Treg cells (iTr35) in mice and humans [22].

Although the immunosuppressive role played by IL-35 on Th1 and Th17 cells has been investigated more [26,39,40,41], the role of IL-35 on Th2 cells was not clearly defined. More importantly, the possible role of IL-35, at doses compatible with those produced by trophoblast cells, on human Th2 cells and also on human Th1, Th17 cells, and Treg cells is completely unknown. In particular, the few investigations performed on the effect of IL-35 on Th2 cells were performed at high concentrations that are not compatible with trophoblast cell production and on murine but not human Th2 cells. In fact, it has been shown that in mice intratracheal delivery of recombinant IL-35 significantly attenuated allergic airway inflammation, probably by reducing the Th2 effector responses [42], and IL-35 at very high concentrations (from 30 to 50 ng/mL) stimulates IL-10 production by murine Th2 cells [30].

Here, we found that rhIL-35, at low concentrations compatible to those produced by first-trimester trophoblast cells (<1 ng/mL), is able to increase the production and the mRNA expression of IL-35, IL-4, and IL-10 by CD4+ T helper cells but has no effect on the production of the other Th2-type cytokines, IL-13 and IL-5, and no effect on the Th1-type cytokine, IFN-gamma, produced by these cells. IL-35 at the same low concentrations decreased the Th17-type cytokine, IL-17A, but has no effect on the other Th17-type cytokine, IL-17F (Figure 1). None of the studies previously published on IL-35’s effects on T cell responses used low concentrations of IL-35. All of them were performed using very high levels of IL-35 (from 10 ng/mL to 100 ng/mL), which are 10- to 100-fold higher than those produced by the human cytotrophoblast cells and HTR8 cells (Figure 5). In fact, we found that the levels of IL-35 measured by a bead-based assay in HTR8 cells and human trophoblast cells supernatants varied but absolutely remained under 1.4 ng/mL (217 ± 15 pg/mL and 1377 ± 278 pg/mL, respectively). The levels of IL-35 produced by trophoblast cells have never been found over 10 ng/mL to 100 ng/mL. These results are compatible with those found by Jia Liu et al. [34], who determined that the content of IL-35 measured in the culture supernatant of HTR8 cells by ELISA was 3857 pg/mL.

The stimulating effect of IL-35 on the CD4+T cell IL-35, IL-4, and IL-10 production and the suppressing effect of IL-35 on T cell IL-17 production seemed to be due to the direct action of IL-35 on CD4+ T cells and not due to the indirect effects of rhIL-35 on APCs, which was unable to decrease IL-23, IL-1beta (Th17 inducer), and IL-12 (Th1 inducer) or to increase IL-10 (Th2 polarization inducer) productions by the APCs presenting the antigen to CD4+ T cells (Figure 1C). Therefore, IL-35 could be a direct inducer of CD4+ T cells producing IL-35, IL-10, and IL-4, although it has been shown that IL-35 promotes M2 polarization [31]. We found that the IL-35+, IL-10+, IL-4+ CD4+ T cells generated in the presence of IL-35 at low doses could be CD4+ Th2-type helper cells producing IL-35 in addition to IL-4 and IL-10 but could also be Foxp3+Tregs, which do not produce IL-4 and IL-10, only IL-35 (Figure 3B), and express p35, EBI3, STAT1, and STAT4, the well-known transcription factors expressed by Treg cells producing IL-35 [43]. Contrary to Liu et al. [34], who asserted that in T cells responsible for maternal–fetal tolerance IL-35 is signaled through STAT1 and STAT3 but not through STAT4, we found that IL-35 induced in T cells the signaling of STAT1 and STAT4. Our results are in agreement with those that demonstrated that the signaling of STAT1 and STAT3 induced by IL-35 is found in B reg cells and not in T cells in which IL-35 has induced the signaling of STAT1 and STAT4 [43].

Moreover, we demonstrated that IL-35, at concentrations compatible to those produced by trophoblast cells, was not only able to induce the differentiation of CD4+ T cells towards an IL-35+, IL-10+, IL-4+ Th2-type functional profile when APCs present the antigen to CD4+ T cells but was also able to modify the cytokine profile of established CD4+T-cell clones, in particular, by increasing the mRNA expression and production of IL-4 and IL-10 but not of IL-35, especially in Th1-type T-cell clones, which acquired the ability to produce IL-4 in the presence of IL-35 (Figure 4). We confirmed these results by showing that the supernatant of purified first-trimester human extravillous trophoblasts containing very low levels of IL-35 (25 pg/mL to 50 pg/mL) but no IL-10 and IL-4 was able to induce the increased production of IL-4 and IL-10 by established CD4+ T helper cell clones (Figure 5).

In agreement with our findings, other authors observed the significant decrease in IL-17A and increase in IL-10 production in isolated human peripheral blood conventional T cells treated with HTR8 supernatant compared with the control group (the experiment with isolated human first-trimester cytotrophoblast cells was not performed by these authors) [34], but contrary to us, they did not observe the effect on IL-4 production and showed a decreased production of IFN-γ by these T cells. The same authors asserted that T cells responding to the modulating effects of IL-35 were iTr35 cells that were able to produce IL-10 and IL-35 [34]. Our results and their results could suggest that IL-35 derived from trophoblast cells could act by increasing IL-10 production on different immunosuppressive cells, Th2 cells and iTr35, respectively, which together are able to participate in the maternal–fetal tolerance in pregnancy. Although these authors asserted that the cells responsible for IL-10 production in response to IL-35 were iTr35 cells [34], Collison et al. [22] described iTr35 cells as cells that suppress exclusively through IL-35 and independently of IL-10 or any other known Treg suppressive mediators (TGFβ and CTLA-4). In fact, iTr35 cells were described as cells that are phenotypically and functionally distinct from the other known induced regulatory populations (Treg cells and Tr1 cells) and are described as cells that exhibit the highly restricted gene signature CD4+ Foxp3− Ebi3+ p35+ IL-10− TGFβ− T cells, excluding IL-10 production [22]. Thus, the role of iTr35 cells on fetal allograft tolerance by producing IL-10 and IL-35 in response to trophoblast IL-35 production has to be clarified.

We found that IL-35, at concentrations compatible with those secreted by purified human first-trimester cytotrophoblast cells, could stimulate the production of IL-35 by regulatory cells, but these regulatory cells did not appear to be iTr35 cells but Foxp3+ EBI3+ p35+ STAT1+ STAT4+ Treg cells, which did not produce IL-4 or IL-10 but only produced IL-35. In agreement with our results, Collison et al. [44] asserted that IL-35 is constitutively secreted by mouse Foxp3+ Tregs.

We also showed that IL-35, at concentrations compatible with those secreted by purified human first-trimester cytotrophoblast cells, could stimulate the production of IL-10, IL-4, and IL-35 by Th2-type CD4+ cells. The fact that IL-10 could be produced by CD4+ T helper cells in response to IL-35 is in accordance with experiments performed in vivo using the model of K14-VEGF-A-Tg mice, which confirmed that IL-10 produced in vivo in response to IL-35 could be produced by Treg cells but also demonstrated that after IL-35 stimulation IL-10 could be produced by CD4+ T helper cells [31]. In vitro murine CD4+ T helper cells were shown to produce IL-10 in response to very high concentrations of IL-35 (from 30 to 50 ng/mL), which are not compatible with those produced by trophoblast cells at the maternal–fetal interface [30].

The suppressing effect of IL-35 on T cell IL-17 production that we observed (Figure 1A and Figure 4C) has also been shown using the same K14-VEGF-A-Tg mouse model [31].

Other authors asserted that IL-35, at higher concentrations than those produced by trophoblasts (10 ng/mL), could suppress grass pollen allergen-driven production of IL-4 and IL-17 and also the production of other Th2-type cytokines, such as IL-5, IL-9, and IL-13, whereas it could induce the expression of IFN-gamma and IL-10 by effector T cells [45]. Our results, which seem to be in contrast to these findings might suggest that the effects of IL-35 on effector T cells could be IL-35-concentration-dependent.

In fact, the concentration of IL-35 also seems to be crucial for the modulating activity of IL-35 on the T cell proliferative response. We found that the concentrations of IL-35 produced by trophoblast cells were not capable of inducing an inhibition of the T helper cell proliferative response, but at very high levels (50 to 100 ng/mL), not compatible with those found in the supernatant of human trophoblast cells, IL-35 could suppress T helper cell proliferation (Figure 6). The effects of high concentrations of IL-35 on T cell proliferation are in agreement with those showing that at very high concentrations (50 and 100 ng/mL) of human recombinant IL-35 the proliferation of stimulated T cells was inhibited [26,34]. Our findings indicate that the levels of IL-35 produced by human trophoblast cells could not play a role in fetal allograft tolerance by directly suppressing effector CD4+ T cell proliferation.

Our results seem to indicate that IL-35, at concentrations secreted by trophoblast cells, by increasing the production of IL-35 by foxp3 Treg cells, by increasing the production of IL-35, IL-4, and IL-10 by CD4+ Th2-type helper cells, and by decreasing IL-17 production by Th17 cells, but not by decreasing the proliferative activity of CD4+ T cells, could contribute to the fetal semi-allograft tolerance and, thus, to the maintenance of pregnancy. It has been shown that IL-35 could have a role in allograft tolerance. In particular, it has been demonstrated that continuous production of IL-35 in mesenchymal stem cells in vivo successfully alleviates cardiac allograft rejection and prolongs the graft survival [33]. The fact that IL-35 could favor the success of human pregnancy has been suggested by studies reporting that the levels of IL-35 decreased in the plasma of women with a history of idiopathic recurrent pregnancy loss compared to fertile controls [35,36] and in the serum and placenta of severe preeclampsia compared with normal pregnant women [46]. More recently, it has been shown in mice that the expression of IL-35 mRNA and protein was downregulated in the placenta of females of the CBA/J × DBA/2 J mating group, which is associated with spontaneous abortion, compared to females of the CBA/J × BALB/C mating group, which is associated with successful pregnancy [34].

We report here the first evidence of the IL-35+, IL-10+, IL-4+ Th2-type CD4+ T-cells that are polarized in response to very low levels of IL-35 that are compatible with those secreted by human trophoblast cells. We showed that the presence of IL-35+, IL-10+, IL-4 + Th2-type CD4+ T-cells is stringently dependent on the presence of an implanted embryo/trophoblast capable of producing IL-35. In fact, IL-35+, IL-10+, IL-4+ Th2-type CD4 + T-cells are present in the deciduae of normal pregnancies where p35 and EBI3 are expressed (Figure 7A) and at the tubal embryo implantation sites of ectopic pregnancies and were completely absent away from the tubal implantation sites and in the deciduae of ectopic pregnancies (where the decidual implantation site is absent but it is present in the fallopian tube) (Figure 7B). These findings strongly suggest that the presence of IL-35+, IL-10+, IL-4+ Th2-type CD4 + T-cells is stringently dependent on the presence of an implanted embryo/trophoblast, which is a source of IL-35 (Figure 5). Thus, the proximity of CD4+ T cells to an IL-35-producing trophoblast/embryo could be a decisive factor for the differentiation of CD4+ T cells towards the IL-35+, IL-10+, IL-4+ CD4+ Th2-type cell phenotype associated with successful pregnancy.

During pregnancy, the fetal allograft tolerance could, in part, depend on a complex relationship between trophoblast cells and decidual immune cells. In fact, trophoblast cells could participate in the recruitment of immune cells such as T cells and in their polarization towards Th2 and Th17/Th2 profiles [14,47]. Here, we show that human first-trimester primary trophoblast cells spontaneously secrete IL-35 and that IL-35 derived from human trophoblast cells might play an important role in the maintenance of maternal–fetal tolerance by polarizing CD4 + T helper cells in Th2-type cells producing IL-35, IL-4, and IL-10 at the embryo implantation site. The role in fetal allograft tolerance of Foxp3+ EBI3+ p35+ STAT1+ STAT4+ Treg cells, shown to be induced by IL-35 at concentrations compatible with those secreted by trophoblasts, is under investigation. Finally, our findings seem to indicate that the suppression of CD4+ T cell proliferation, in response to the low levels of IL-35 produced by human trophoblasts, is not the mechanism responsible for fetal allograft tolerance.

## 4. Material and Methods

### 4.1. Reagents

PHA was purchased from GIBCO Laboratories (Grand Island, NY, USA) and phorbol 12-myristate 13-acetate (PMA) was purchased from Sigma Chemical Co. (St. Louis, MO, USA). OKT3 (anti-CD3) mAb was purchased from Ortho Pharmaceuticals (Raritan, NJ, USA). Anti-CD4 monoclonal antibodies (mAb) and anti-CD8 mAbs were obtained from Becton–Dickinson (Mountain View, CA, USA). Human recombinant IL-2 was a generous gift of Eurocetus (Milano, Italy). FCS was from HyClone Lab Inc. (Logan, UT, USA). SK was obtained from Sanofi Aventis (Paris, France). RhIL-35 was purchased from R&D Systems, (Minneapolis, MN, USA).

### 4.2. Generation of Antigen-Specific T-Cell Lines in the Absence or Presence of Recombinant Human IL-35 (rhIL-35)

Streptokinase (SK)-specific T-cell lines were generated from five donors, as described elsewhere [48]. Briefly, 10^6^ peripheral blood mononuclear cells (PBMC) in 2 mL of RPMI 1640 medium supplemented with 2 mM L-glutamine, 2 × 10^−5^ M 2-mercaptoethanol (complete medium), and 2.5% human serum were stimulated in 24-well flat-bottomed plates for 5 days with the SK antigen (500 UI/mL) in the absence or presence of rhIL-35 (300 pg/mL) and, as controls for cytokine modulation, in the presence or absence of recombinant IL-12 (R&D System, 5000 pg/mL). Human IL-2 at 20 U/mL was then added and cultures continued for an additional 9 days.

The generated T-cell lines were tested for their SK specificity as follows: 2 × 10^4^ viable T blasts from SK-specific T-cell lines were seeded in microplates and co-cultured for 48 h with irradiated (9000 rad) autologous PBMC (5 × 10^4^) in the presence of medium alone or SK (100 UI/mL). After a 16 h pulse with 0.5 µCi 3H-TdR (Amersham, UK), the cultures were harvested, and the radioactivity was measured by liquid scintillation.

To induce cytokine production by T-cell lines, 10^6^ T blasts from each SK-specific T line generated in the absence or in the presence of rhIL-35 and in the presence of IL-12 were cultured in the presence of PMA (Sigma, 20 ng/mL) plus anti-CD3 monoclonal antibodies (BD, 100 ng/mL). After 36 h, culture supernatants were collected and stored in aliquots at −80 °C until used. IL-4, IL-5, IFN-γ, IL-17A, IL-17F, IL-22, IL-10, IL13, and IL-35 were quantified by a multiplex bead-based assay. Values of the cytokine content 5 SD over those of the control supernatants obtained by the stimulation of irradiated feeder cells alone were considered to be effective secretions.

### 4.3. Generation of Antigen-Specific T-Cell Clones Derived from Antigen-Specific T-Cell Lines Generated in the Absence or Presence of rhIL-35

To generate T-cell clones from antigen-specific T-cell lines generated in the absence or presence of rhIL-35, T blasts from SK-specific lines obtained from two different donors were seeded under limiting dilution conditions (0.3 cell/well) in six round-bottom microwell plates containing 10^5^ irradiated (6000 rad) allogeneic PBMC cells (as feeder cells) and PHA (1% *v*/*v*) in a final volume of 0.2 mL of complete medium supplemented with IL-2 (20 UI mL) and 10% FCS, as reported elsewhere [48]. Growing microcultures were then supplemented, at weekly intervals, with IL-2 (20 U/mL) and 10^5^ irradiated feeder cells. The phenotype distribution of the T-cell clones was assessed by flow cytometer analysis. The SK specificity of the T-cell clones was assessed by measuring the ^3^H-TdR uptake after 6 h of stimulation with SK under MHC-restricted conditions [48]. When the stimulation index (the ratio between the mean counts per minute obtained in the cultures stimulated with APC plus SK and the mean counts per minute obtained in the cultures with APC alone) was >10, the responses were considered to be positive.

### 4.4. Generation of T-Cell Clones Derived from Peripheral Blood

To generate T-cell clones from peripheral blood, peripheral blood mononuclear cells (PBMCs) from four normal subjects were seeded under limiting dilution conditions (0.3 cell/well) in six round-bottomed microwell plates containing 10^5^ irradiated (9000 rad) allogeneic PBMC (as feeder cells) and PHA (1% vol/vol) in a final volume of 0.2 mL of complete medium supplemented with IL-2 (50 U/mL) and 10% FCS, as reported elsewhere [48]. Growing microcultures were then supplemented, at weekly intervals, with IL-2 (50 U/mL) and 105 irradiated feeder cells. The phenotype distribution of the T-cell clones was assessed by flow cytometer analysis.

### 4.5. IL-4, IL-10, IFN-γ, IL-17A, Foxp3, EBI3, p35, GATA3, T-bet, and ROR-C mRNA Expression of Antigen-Specific T-Cell Lines and CD4+ T-Cell Clones

Total RNA was extracted with an RNAsy Kit and treated with DNase I (Qiagen, Hilden Germany) from antigen-specific T-cell lines, CD4+ T-cell clones derived from antigen specific T-cell lines, normal peripheral blood, and decidual tissue from a normal pregnancy. cDNA was synthetized using TaqMan Reverse Transcription Reagents (Applied Biosystem, Warrington, UK). Real Time Polymerase Chain Reaction (RT-PCR) was then performed by using the TaqMan methodology as described elsewhere [49]. A quantitative analysis of IL-4, IL-10, IFN-γ, IL-17A, Foxp3, EBI3, p35, GATA3, T-bet, ROR-C, and β-actin was performed using Assay on Demand (Applied Biosystem, Warrington, UK). The concentration of the extracted RNA was evaluated using a NanoDrop ND-1000 spectrophotometer (Thermo Scientific, DE, USA). The RNA concentrations varied from 102 to 300 ng/microliter for the T cells and from 1324 to 1590 ng/microliter for the decidual tissues. The integrity of the RNA was evaluated by agarose gel electrophoresis. Beta-actin was used for normalization. Beta actin was chosen as the housekeeping gene based on preliminary experiments that were performed to evaluate the reliability of different housekeeping genes, such as GAPDH, PPIB, HPRT1, and beta-actin, on T-cell lines, T-cell clones, and decidual tissues and based on the literature [50,51,52,53], which demonstrated the stability and suitability of beta-actin as a reference gene for these cells and specimens.

### 4.6. Generation of CD4+ T-Cell Clones from Decidual Tissue of First-Trimester Normal Pregnancy and from Fallopian Tube and Decidual Biopsies of Ectopic Pregnancy

Four pregnant women with normal gestation and no spontaneous abortion in their past history requested elective termination and were enrolled in our study. The four specimens of decidua were obtained at 11 weeks of pregnancy. Trophoblast-invaded tubal mucosa at the implantation site, tubal mucosa distant from the implantation site, and a decidua specimen without any implantation site were obtained from a woman (with no spontaneous abortion in her past history) whose ectopic pregnancy was terminated by surgical removal as a result of a threatened tubal rupture. The women agreed to participate to the study at the Hospital of Rijeka, Croatia. They received verbal and written information about the aim and the design of the research. All the women signed the informed consent, and the study was approved by the local ethics committees of the Medical Faculty of Rijeka of the Clinical Hospital Center of Rijeka (N. 2170-29.02/1-06-1 and 2170-24-09-7-06-02).

Specimens of deciduae and of fallopian tube biopsies were washed twice in PBS (pH 7.2) and then disrupted in small fragments (2−3 mm in diameter). Short-term T-cell lines were generated by culturing single fragments for one week in 24-well plates (Costar, Cambridge, MA, USA) in 2 mL of complete medium supplemented with 10% FCS and IL-2 (20 U/mL) to allow only T cells activated in vivo to grow. T-cell clones were then generated from short-term cultures of decidual and tubal T cells derived in the presence of IL-2 using a method described elsewhere [7]. The phenotype of CD3+, CD4+ T-cell clones was assessed by flow cytometer analysis.

### 4.7. Induction of Cytokine Production by Antigen-Specific T-Cell Clones Derived from Antigen-Specific T-Cell Lines Derived from Peripheral Blood and Decidual and Tubal Biopsies

To induce cytokine production, 10^6^ T-cell blasts from each T-cell clone were cultured in the presence of PMA (20 ng/mL; Sigma, St. Louis, MO, USA) plus a monoclonal antibody against CD3 (100 ng/mL; Ortho Pharmaceuticals, Raritan, NJ, USA). After 36 h, culture supernatants were collected, filtered, and stored in aliquots at −80 °C.

### 4.8. Determination of Cytokine Concentrations in Supernatants of Antigen-Specific T-Cell Lines, of T-Cell Clones Derived from T-Cell Lines, of T-Cell Clones Derived from Peripheral Blood and from Decidual and Tubal Biopsies, of Peripheral Blood Monocytes, and of Trophoblast Cells and HTR8 Cell Lines with Bead-Based Multiplexed Immunoassays

The quantitative determination of IL-4, IL-5, IL-13,IL-10, IL-17A, MIP-3α, IL-1-β, IL-23, IL-6, IL-12, IL-27, TNF-α, and IFN-γ was performed by a multiplex bead-based immunoassay (Biorad Laboratories, Hercules, CA, USA), and the determination of IL-35 was performed by another bead-based immunoassay (Millipore, Billerica, MA, USA) using a Bioplex 200 system (Biorad Laboratories, Hercules, CA, USA), as we have described elsewhere [54]. In brief, supernatant was added to antibody-conjugated beads directed against the cytokines listed above in a 96-well filter plate. After a 30 min incubation, the plate was washed, and a biotinylated anti-cytokine antibody solution was added before another 30 min incubation. The plate was then washed, and streptavidin-conjugated PE was added. After a final wash, each well was suspended with assay buffer and analyzed with the Bioplex 200 system. Standard curves were derived from various concentrations of the different cytokine standards and followed the same protocol as the supernatant samples. The concentration of each cytokine (pg/mL) in each supernatant was calculated thanks to the Bioplex200 software.

### 4.9. Proliferation of Peripheral Blood CD4+ T-Cell Clones in Response to rhIL-35

Viable T blasts (10^5^) derived from nine CD4+ T-cell clones derived from the peripheral blood of healthy donors were stimulated in microplates with immobilized anti-CD3 monoclonal antibodies for 48 h in the presence of complete medium supplemented with FCS 10% alone or in the presence of complete medium supplemented with FCS 10% and rhIL-35 at different concentrations (from 300 pg/mL to 100 ng/mL). After a 16 h pulse with 0.5 µCi 3H-TdR (Amersham, UK), cultures were harvested, and radioactivity measured by liquid scintillation.

### 4.10. Induction of Cytokine Production of Macrophages Stimulated by SK in the Absence and Presence of rhIL-35

Monocytes (10^6^) that were obtained from five donors and were previously purified (mean ± SD; 92.59 ± 2.51%) by adherence from PBMCs in 1 mL of complete medium in 96 U-bottomed plates for 5 days with the antigen SK (10 µg/mL) in the absence or in the presence of rhIL-35 (300 pg/mL). After 5 days, the supernatants were collected and stored in aliquots at –80 °C until used.

### 4.11. Isolation of Human First-Trimester Cytotrophoblast Cells

Placental specimens were obtained from women undergoing elective termination of pregnancy at 8–12 weeks of gestation. The study was approved by the institutional review board of The Maternal-Children Hospital (IRCCS ‘Burlo Garofolo’, Trieste, Italy), and informed consent was obtained from all women providing tissue specimens. The CTBs were isolated as previously described with some modifications [55]. Briefly, fragments of placental tissue containing floating and anchoring villi were finely minced and incubated with Hanks balanced salt solution (HBSS; GIBCO, Invitrogen, MA, USA) containing 0.25% trypsin and 0.2 mg/mL DNAse type I (Roche, Milano, Italy) for 20 min at 37 °C. After Percoll gradient fractionation, the contaminating leukocytes were removed by immunomagnetic beads coated with mAb to CD45 (Dynabeads M-450, Invitrogen, Milano, Italy) and cultured on fibronectin (Roche, NY, USA) in RPMI 1640 medium supplemented with 10% fetal bovine serum (FBS) (GIBCO, Invitrogen, MA, USA). Under these conditions, non-adherent leucocytes and syncitiotrophoblasts were removed. The cells were 95% positive for cytokeratin 7, and of these 70% were HLA-G positive extravillous trophoblasts, and 30% were HLA-G negative villous trophoblasts. No remaining leukocytes were detected in our preparations, as assessed by RT-PCR with CD45 primers (data not shown).

### 4.12. Trophoblast Cell Line

The HTR-8/SV neo trophoblast cell line, produced by the immortalization of HTR-8 cells with SV40 virus from primary cultures of CTBs [56], was provided by Peeyush K. Lala (Dept. of Anatomy and Cell Biology, University of Western Ontario, Canada). The cells were grown in RPMI 1640 medium supplemented with 10% fetal bovine serum (GIBCO, Invitrogen, MA, USA).

### 4.13. Culture of Peripheral Blood CD4+ T-Cell Clones in the Presence of Supernatants Derived from Isolated Human Trophoblast Cells

Fourteen CD4+ T-cell clones derived from the peripheral blood of four different healthy donors were stimulated with immobilized anti-CD3 monoclonal antibodies in the presence of the supernatants derived from four different isolated human trophoblasts in which the levels of IL-35 were previously evaluated by a multiplex bead-based assay. The final concentrations of IL-35 derived from trophoblasts placed in the cultures of the CD4+ T cell clones were 25 pg/mL and 50 pg/mL, respectively.

### 4.14. Statistics

Statistical analyses were performed using SPSS software (SPSS, Inc., Evanston, IL, USA). The data were analyzed with normality tests to assess whether the distribution of the data was normal or not. We applied the Kolmogorov and Smirnov test. A small *p*-value (<0.05) implied that the data were not normally distributed. Accordingly, a non-parametric statistical evaluation test was applied. Due to the non-parametric distribution, all comparisons between cytokine concentrations were performed with a Wilcoxon test. A *p* value of <0.05 was considered to be statistically significant.

## Data Availability

Not applicable.
